# High expressions of the cytoglobin and PGC-1α genes during the tissue regeneration of house gecko (*Hemidactylus platyurus*) tails

**DOI:** 10.1186/s12861-020-00214-4

**Published:** 2020-05-11

**Authors:** Titta Novianti, Vetnizah Juniantito, Ahmad Aulia Jusuf, Evy Ayu Arida, Mohamad Sadikin, Sri Widia A. Jusman

**Affiliations:** 1 Doctoral Biomedical Program, Faculty of Medicine, Universitas Indonesia, Kota Depok, Indonesia; 2 Biotechnology Department, Universitas Esa Unggul, Jakarta, Indonesia; 3 Department of Veterinary Clinic Reproduction and Pathology, Faculty of Veterinary Medicine, Agriculture Institute of Bogor, Bogor, Indonesia; 4 Department of Histology, Faculty of Medicine, Universitas Indonesia, Kota Depok, Indonesia; 5 Indonesian Institute of Sciences (LIPI), Cibinong, Bogor, Indonesia; 6 Center of Hypoxia and Oxidative Stress Studies (CHOSS), Department of Biochemistry & Molecular Biology, Faculty of Medicine Universitas Indonesia Jl, Salemba Raya no. 6 Jl, Jakarta Pusat, Indonesia

**Keywords:** Cytoglobin, PGC-1α, Mitochondrial biogenesis, House gecko, Tissue regeneration

## Abstract

**Background:**

The tissue regeneration process requires high oxygen and energy levels. Cytoglobin (Cygb) is a member of the globin family, which has the ability to bind oxygen, plays a role in dealing with oxidative stress, and carries oxygen into the mitochondria. Energy production for tissue regeneration is associated with mitochondria—especially mitochondrial biogenesis. The peroxisome proliferator-activated receptor-gamma coactivator (PGC)-1alpha protein helps to regulate mitochondrial biogenesis. House geckos (*Hemidactylus platyurus*) are reptiles that have the ability to regenerate the tissue in their tails. House geckos were selected as the animal models for this study in order to analyze the association of Cygb with oxygen supply and the association of PGC-1α with energy production for tissue regeneration.

**Results:**

The growth of house gecko tails showed a slow growth at the wound healing phase, then followed by a fast growth after wound healing phase of the regeneration process. While Cygb mRNA expression reached its peak at the wound healing phase and slowly decreased until the end of the observation. PGC-1α mRNA was expressed and reached its peak earlier than Cygb.

**Conclusions:**

The expressions of both the Cygb and PGC-1α genes were relatively high compared to the control group. We therefore suggest that Cygb and PGC-1α play an important role during the tissue regeneration process.

## Background

Tissue regeneration is a complex process that attempts to restore organ morphology to functional levels after an injury. This process involves cell proliferation, migration, and differentiation, and synthesis of the extracellular matrix [1–5]. Since the tissue regeneration process aims to restore tissue morphology and physiology, it requires high levels of energy, which in turn require the aerobic metabolism to produce high numbers of ATPs. The ATPs fulfill their function in this process only when the O_2_ supply is sufficient [5–7]. If the aerobic metabolism does not keep pace with the increase of oxygen demand, the tissue enters a relative hypoxia state [8, 9]. In this state, the organisms strive to meet the oxygen demand in order to maintain the metabolism for cellular activities; hence, oxygen might be transferred to the mitochondria by the Cygb protein [10–12].

The high affinity of Cygb to oxygen led to our assumption that Cygb might play a significant role as an oxygen diffusion factor, transferring oxygen to the mitochondria. Cygb protein also has a function in oxygen storage and as an oxygen sensor [13, 14]. This functionality is similar to that of the myoglobin in muscle cells, which contributes to the maintenance of the oxidative phosphorylation process [15, 16]; therefore, we suspected that Cygb was involved as an adaptive response to hypoxia-mediated injuries.

Mitochondria, as energy-producing organelles, play an important role in certain cellular events, such as cell proliferation and differentiation, which require high energy levels. When these cells demand high levels of energy, the mitochondrial biogenesis process meets the energy demand increase [6, 17, 18]. PGC-1α is a well-known mitochondrial biogenesis biomarker and its presence in the cell nucleus regulates the nuclear respiratory factor (Nrf-1) and mitochondrial transcription factor A (mtTFA) genes. Both genes play a role in regulating mitochondrial biogenesis [6, 18, 19].

Studies of the gene expression, protein synthesis, and cell structure involved in tissue regeneration during hypoxia are limited, but such medical research is important for analyzing the various aspects of hypoxia during the tissue regeneration process [20, 21]. We used the house gecko (*Hemidactylus platyurus*) as an animal model, because of its ability to regenerate its tail tissue after autotomy. Another reason was that, among the vertebrate animal groups with tissue regeneration ability, the house gecko has the closest taxonomy to mammals [22, 23].

We analyzed the expressions of Cygb and PGC-1α in the tissue regeneration of house gecko (*Hemidactylus platyurus*) tails. We hypothesize that Cygb and PGC-1α expression increase significantly during the tail regeneration process due to the high O_2_ and energy demand. We also hypothesize that Cygb and PGC-1α play a role in the tissue regeneration process.

## Results

### Tail growth

**Fig. 1:**
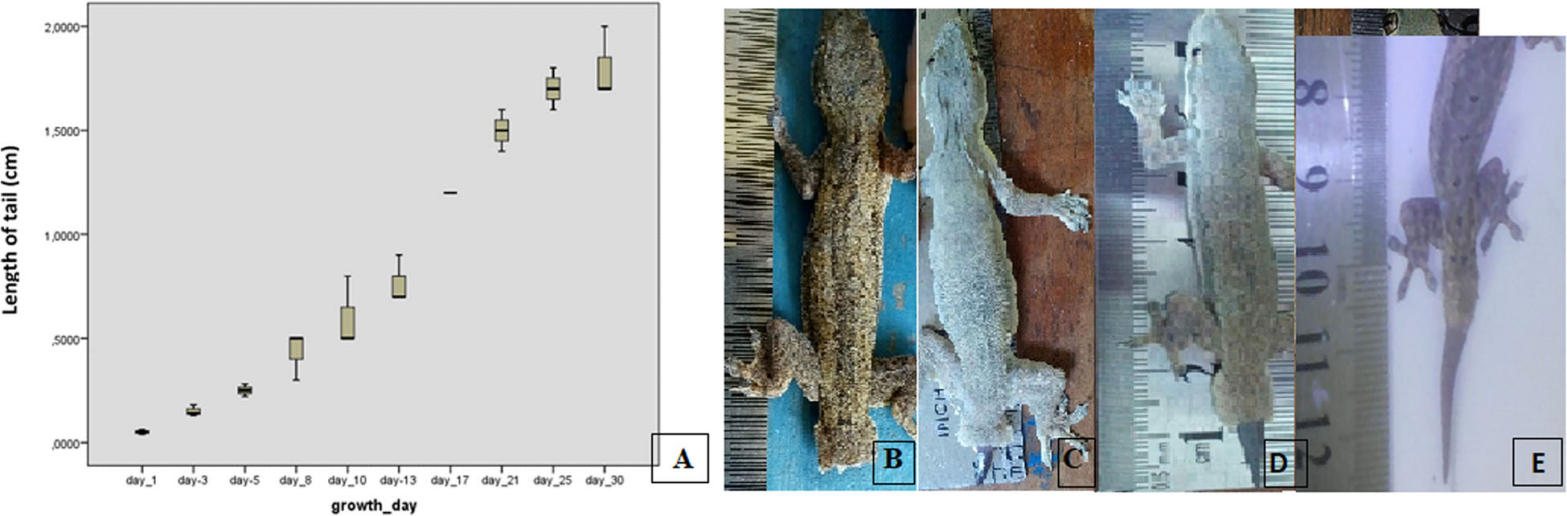
The growth of the house gecko (*Hemidactylus platyurus*) tails. **a** the growth curve of the tail (cm) from day 1 to day 30 (*), indicating significantly different growth between day 13 and day 17, and between day 17 and day 21 (*p* < 0.05, Kruskal-Wallis test); **b** the length of the house gecko tail on day 1 after autotomy and **c** on day 13, **d** on day 17, and **e** on day 21

The growth of the house gecko tails, as a result of the tissue regeneration process, was measured in centimeters from the proximal to the distal parts of the tails. The tails grew slowly from day 1 to day 13 (the wound healing and blastema phase), then the growth curve increased markedly from day 13 to day 21 (the regeneration phase), and increased slowly again from day 21 to day 30 (the maturation phase). The growth of house gecko tails differed significantly between day 13 and day 17, and between day 17 and day 21 in autotomized groups (*p* < 0.05, Kruskal-Wallis test) (Fig. 1a). The differences in tail length on days 1, 13, 17, and 21 are shown in Fig. 1b-e.

### Histological analysis of tail tissue regeneration

**Fig. 2:**
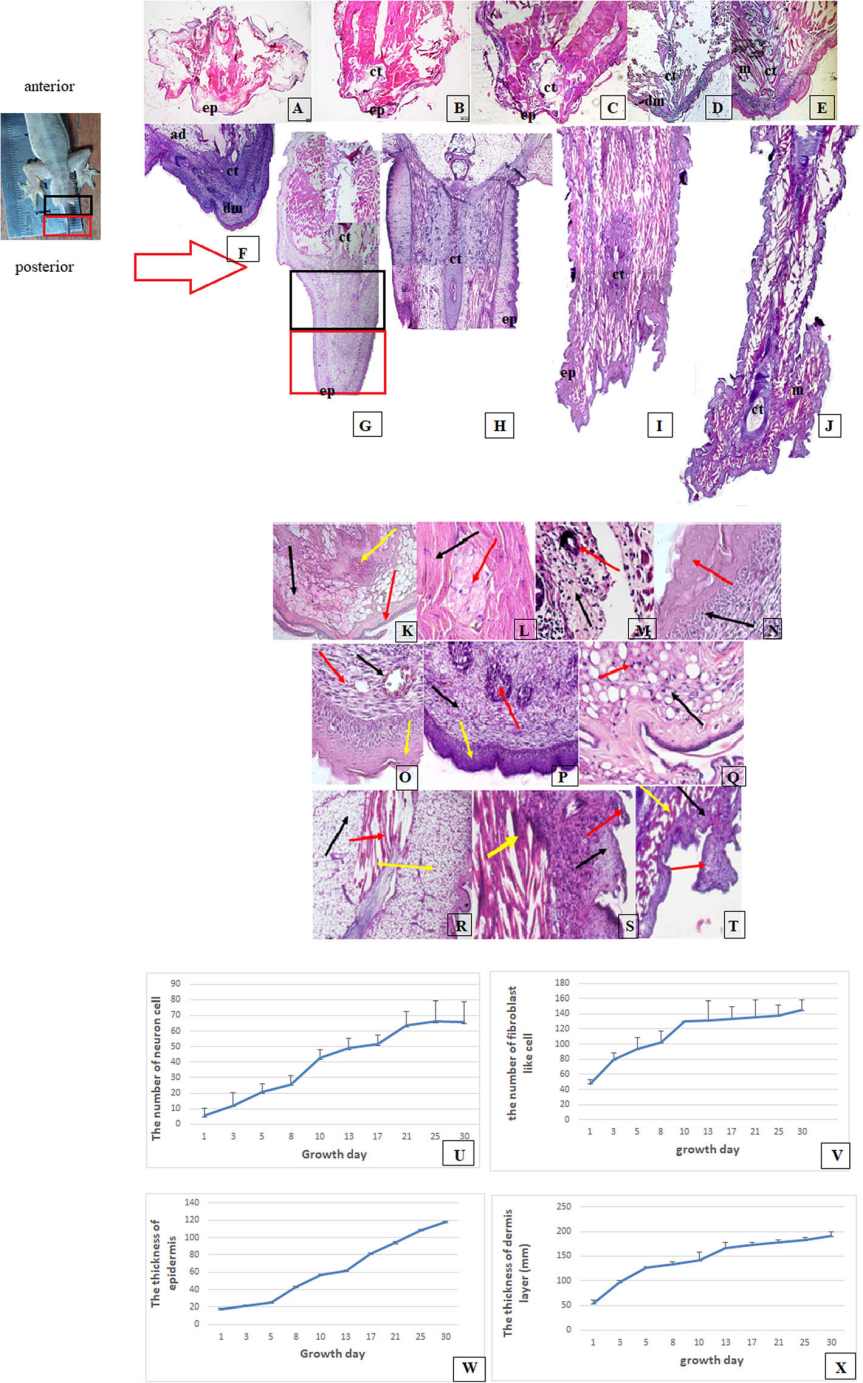
Histological analysis of the tissue regeneration of the house gecko tails: **a**–**j**. comparisons of the tissue growth of the house gecko tail tissue with that of the control, from day 1–30; **a** day 1, **b** day 3, **c** day 5, **d** day 8, **e** day 10, **f** day 13, **g** day 17, **h** day 21, **i** day 30, and **j** control. The growth of the epidermis (**ep**), dermis (**d**), muscle (**m**), and cartilage (**ct**) tissues differed in each phase of tissue regeneration: **k**–**t** tissue regeneration of the house gecko tail; **k** on day 1, an epithelial layer (red arrow) formed and closed the wound—adipose tissue (black arrow) is shown above the dermis layer and cartilage tissue (yellow arrow) is near the adipose tissue; **l** the neuronal ganglion cells (red arrow) formed on day 3, surrounded by muscle tissue (black arrow); **m** on day 5, the blood vessels (red arrow) formed in the dermis (black arrow); **n** on day 8, the epidermal layer (red arrow) was thicker and the progenitor cell spread in the basal lamina (black arrow); **k**–**n** were the histological analyses in the wound healing phase; **o** on day 10, the blood vessels (black arrow) enlarged and spread in the dermis (red arrow), the epidermis layer (yellow arrow) was thicker, and the basal lamina (blue arrow) was enriched with progenitor cells; **p** on day 13, the blastema cells (red arrow) aggregated in the connective tissue (black arrow), and the epidermis layer (yellow arrow) was thicker (days 10 to 13 were the blastema phase); **q** on day 17, the new blood vessels (black arrow) spread in the connective tissue (red arrow) (days 13–17 were the regeneration phase); **r** on day 21, the new adipose tissue (black arrow) and the new muscle tissue (red arrow) appeared in the regenerating tail tissue, and the epidermis (yellow arrow) grew thicker; **s** on day 30, the epidermis (black arrow) and new muscle (red arrow) became more compact and scales appeared on the epidermis layer (days 21–30 were the maturation phase;) **t** for the control—the non-autotomized tail tissue of the house gecko**—**the scale (red arrow), the dermis (black arrow), and the muscle (yellow arrow) were more compact, at a magnification of 40 × 10. **u**–**x** The graphs of the quantitative histological analyses of the regenerated house gecko tail tissue: **u** the density (/mm^2^) of the neuronal cells in regenerated tail tissue; **v** the density (/mm^2^) of the fibroblast-like cells in the regenerated tail tissue; **w** the thickness (mm) of the epidermis of the regenerated tail tissue; **x** the thickness (mm) of the dermis layer of the regenerated tail tissue

The histology of the all the regenerated tail samples and the control samples, from day 1 to day 30, is shown in Fig. 2a–j. The growth of the epidermis, dermis, muscle, and cartilage tissues differed for each phase of the house geckos’ tissue regeneration. The epidermis and dermis tissues grew thicker during the tissue regeneration, and the muscle and cartilage tissues grew more compact to form the new tail. On day 30, scales appeared at the edge of the epidermis.

The histological analysis for each day of growth is shown in Fig. 2k–t. The wound-healing and the remodeling phase lasted from day 1 to day 10, and the cells actively proliferated, differentiated, and migrated during this period. In this phase, the epithelial layer formed and closed the wound, and the fibroblasts and neuronal ganglion cells spread in the dermis. The formation of the endothelial cells of the blood vessels in the endodermis occurred on day 5, and the endothelial cells enlarged on day 8, with the epidermis and basal lamina cells growing and becoming thicker. On day 10, stem cells containing granules appeared in the dermis, and new blood vessels appeared in some tissues (Fig. 2k- o). The high activity of the cells is shown by the curves for cell density and tissue width in Fig. 2u–x.

The tissue regeneration, which entered the blastema phase on day 13, indicated that an aggregation of stem cells occurred, and the epidermis and dermis tissues grew more compact. Days 17 to 21 were the regeneration phase, during which some of the new tissues formed. The blood vessels grew rapidly to deliver the blood supply to the tissues, and new adipose and muscle tissues appeared in the dermis. The epidermis, dermis, and connective tissues also grew rapidly (Fig. 2q–r).

In the maturation phase, the adipose, muscle, epidermis, dermis, and connective tissues grew rapidly and became more compact. Scales formed at the edge of the epidermis of the regenerated house gecko tails on day 30 after autotomy (Fig. 2s). The histology of the regenerated tissue on day 30, as well as that of the control group (which had not undergone autotomy) are shown in Fig. 2t.

### Immunohistochemical (IHC) analysis of the Cygb protein

**Fig. 3:**
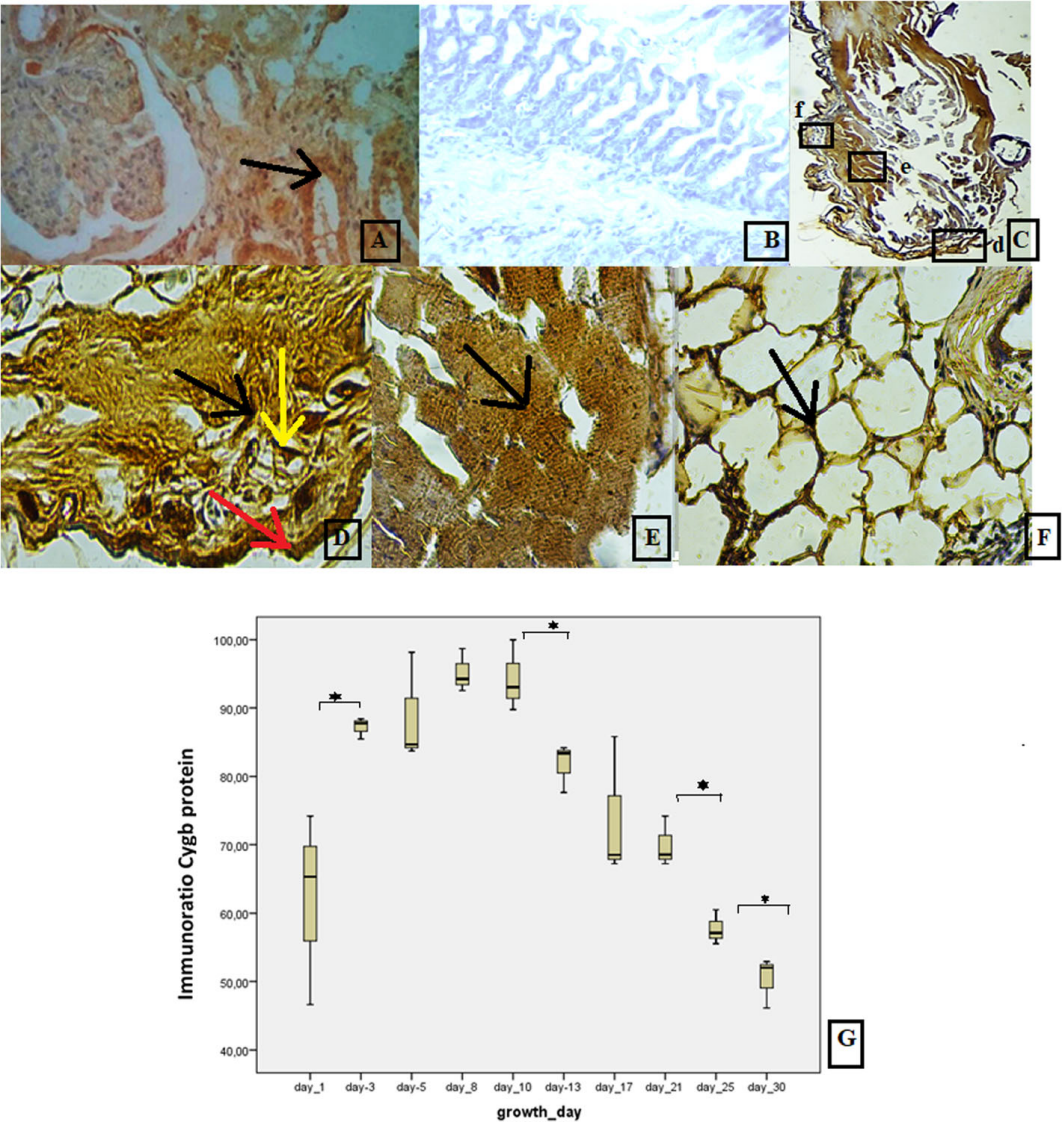
IHC Analysis of Cytoglobin protein: **a–f** IHC analysis of Cygb protein in the regenerated tissues of house gecko tails; **a** positive control using a section of mouse small intestine, with the arrowhead pointing to the Cygb protein in the mucosa cells (darker brown), with the rest of the brown staining in the background; **b** negative control using a sample obtained from house gecko tail tissue that did not undergo autotomy; **c** the whole regenerated house gecko tail tissue on day 3; **d**. darker brown staining showing the expression of Cygb in fibroblast-like cells (black arrow), in the polygonal shape (yellow arrow) that characterized a neuronal cell, and in epidermal cells (red arrow), with the rest of the brown staining in the background; **e** Cygb spread in intracellular of muscle cells; **f** Cygb spread in the adipose tissue (black arrow) and through the epithelial cells (double arrow), at a magnification of 40 × 10); **g** box plot of Cygb protein expression based on quantitative analysis using ImmunoRatio from day 1 to day 30, showing significant differences between day 1 and day 3, between day 10 and day 13, and between day 21 and day 25

Cygb protein spread in the epidermis, neuronal cells, adipose tissues, fibroblast-like cells, and muscle cells (Fig. 3d–f). The whole of the regenerated tail tissue on day 3 is shown in Fig. 3c. The Cygb protein from day 1 to day 30 was quantitatively analyzed using **I**munoRatio. The ImmunoRatio box plot is shown in Fig. 3g and there were several different expressions between day 1 and day 3, between day 10 and day 13, between day 21 and day 25, and between day 25 and day 30.

### Expressions of Cygb and PGC-1α mRNA

**Fig. 4:**
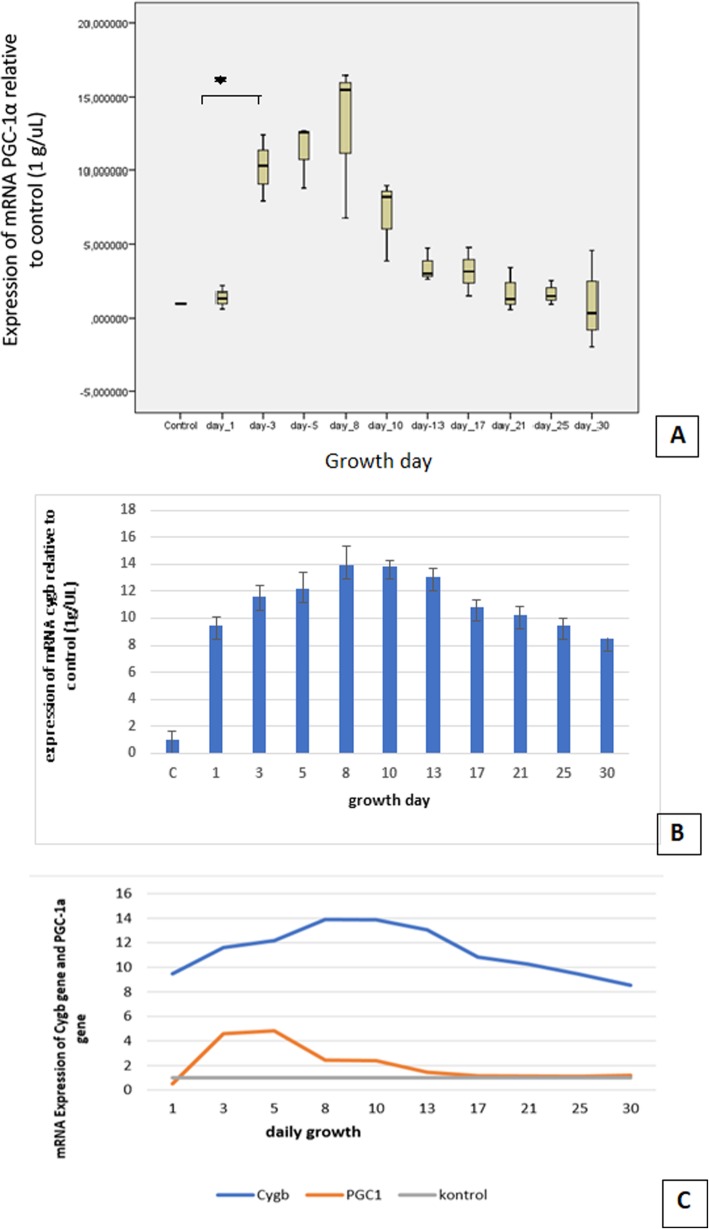
Analysis of the Cygb and PGC-1α expressions in regenerated house gecko tail tissues: **a** the box plot of mRNA PGC-1α expression relative to the control group (C) from day 1 to day 30 **(***n* = 33), during which period the data distribution was not normal (*p* < 0.05) and the data differed significantly between growth days 1 and 3 according to the non-parametric Kruskal-Wallis test (*p* < 0.05); **b** the graph of Cygb mRNA expression from day 1 to day 30 (*n* = 33; mean ± SEM) during which period the data distribution was normal, (*p* > 0.05), and the data varied between growth days according to the parametric ANOVA (*p* < 0.05) **c** comparison of the linear curves for the gene expression of Cygb and PGC-1α from day 1 to day 30 (*n* = 33, mean ± SEM), during which period the data distribution was normal (*p* > 0.05), and the data varied between growth days according to the parametric ANOVA (*p* < 0.05). The Cygb expression was high during the tissue regeneration process; the PGC-1α expression was higher from day 3 to day 5, decreased by day 8, but was still higher than that of the control group during the tissue regeneration process

The expression of PGC-1α on day 1 was relatively low, but increased sharply during days 3 to 5. From day 8, the expression decreased slowly until day 30, at which point the expression of the PGC1-α gene was still slightly higher than that of the control group (*p* < 0.05, Kruskal-Wallis test) (Fig. 4a). We identified the data variation for each group using the Mann-Whitney test (Table S2 in Additional file 1).

The expression of Cygb mRNA was high relative to the control group during the tissue regeneration of the house gecko tails from day 1 to day 30. It reached a peak on day 8, then decreased slightly until day 30; however, the Cygb expression was still higher than in the control group (Fig. 4b). We observed that the expression of Cygb mRNA differed significantly on each growth day (*p* < 0.05, ANOVA) and there are different significantly between day 13 and day 17 post amputation of post hoc analysis.

The compared expression curves of the Cygb and PGC1-α genes showed different patterns. The expression of the PGC-1α gene reached a peak on day 3, while the peak expression of the Cygb gene occurred on day 8. The expression of Cygb was higher than the expression of the PGC-1α gene during the tissue regeneration process (Fig. 4c).

### Quantitative histological analysis

The quantitative histological analysis using the Image-J program counted the density of the fibroblast-like cells and neuronal cells. The thickness of the dermis and epidermis tissues were measured using the ImageJ program, which presented the width of the tissues at mm scale. Data was collected from five visual fields for one slide preparation with a magnification of 40 × 10.

The curves for the cell density of fibroblast-like cells and neuronal cells are shown in Fig. 2u-v. The results of the Kruskal-Wallis test for fibroblast-like cell density showed a significant difference between day 10 and day 13 (*p* < 0.05), and a further significant difference between day 1 and day 3 for the density of the neuronal cells.

The thickness of the dermis and epidermis tissues are shown in Fig. 2x–y. The thickness of the epidermis layer differed between day 5 and day 8, between day 13 and day 17, and between day 8 and day 10. The dermis thickness differed significantly between day 1 and day 3, and between day 10 and day 13. Both of the quantitative analyses used the Kruskal-Wallis test (p < 0.05).

## Discussion

Basic molecular biological research regarding the tissue regeneration process is vitally important, but studies of the role played by Cygb and PGC-1α gene expressions in the tissue regeneration process of animals are limited [18, 24, 25]. We used the house gecko (*Hemidactylus platyurus*), from the Gekkonidae family, as an animal model, because of its ability in autotomize and regenerate its tail. Furthermore, the taxonomy of the house gecko, is the closest to mammals of all the animals that have tissue regeneration ability [22, 26] The house gecko was therefore an appropriate animal model for tissue regeneration research.

It takes 30 days for house gecko tail tissue to regenerate; however, the complete morphogenesis process continues until the tenth week after autotomy. The ability of a house gecko to regenerate its tail is similar to that of a lizard, which forms a complete tail that is ready for the next autotomy in 25–30 days [26, 27]. The tissues of the house gecko tail consist of epithelial, dermis, muscle, bone, nerve, connective, blood, and adipose tissues, as shown on day 30 of our observation (Fig. 2.I and 2.S).

The growth of house gecko tails followed a different pattern at different phases of the regeneration process: in the first 13 days it was relatively slow; from day 13 to day 21 it increased significantly; and from day 2 to day 30, it slowed again. The three phases in the regeneration of the house gecko tail were the wound healing phase, the generation phase, and the maturation phase, [28] and these three distinct phases indicated that there were differences in the related cell and tissue activity.

The first 13 (wound healing) days of the tissue regeneration process were indicated by high cell activity. The characteristics of this phase were cell migration, proliferation, and differentiation. This phenomenon was demonstrated in our study by the increase in the thickness of the dermis layer during this period and the increased quantity of fibroblast-like cells (Fig. 2u–y). This was consistent with Mescher, who stated that the wound healing phase occurs at the beginning of the regeneration process and is characterized by the proliferation, migration, and differentiation of macrophages, fibroblasts, and progenitor cells [29]. In our study, it appeared that various cells actively proliferated, migrated, and differentiated during this wound healing phase. Basal lamina cells in the epidermis layer actively migrated and differentiated to form new epithelial and dermis tissues. Cells such as fibroblasts, neuronal cells, and lymphocyte cells spread in the dermis tissue and differentiated to form new tissues. The high activity of cells in this period had an impact on oxygen and energy demand. In the regenerated tissue of the house gecko tails, no blood existed to distribute oxygen in the wound healing phase. The high Cygb expression in the wound-healing phase indicated that this was an attempt to supply oxygen to the tissues by increasing Cygb expression. We assumed that the increased energy requirements for cell activity in this phase caused an increase in oxygen demand for metabolic processes to produce energy.

In the second phase of the tissue regeneration process, new tissue started to form. According to Alibardi, in the second phase of tissue regeneration, granulation cells appeared that contained stem cells [28]. The stem cells appeared to spread in the dermis (Fig. 2p). These stem cells played a role in the formation of new tissues, such as for the muscles, bones, blood vessels, nerves, and dermis [30]. Cygb expression was still maintained at a higher level than in the control group during this process of supplying oxygen to the regenerating tissue.

The third phase of the tissue regeneration process was the period during which new tissue formed. In this period, the spinal cord tissue elongated, resulting in significant growth of the house gecko tails. Morphogenesis of the new tail tissue began in the maturation phase of tissue regeneration. Thorn-shaped tissue formed on the tail skin to create a pointed tail, and the growth curve of the tail increased only slowly during this period.

The expression of the Cygb gene increased slowly during the regeneration of the gecko tail tissue. When Cygb expression reached its peak, the activity of fibroblast-like cells and neuronal cells was high, as shown in the quantitative data drawn from the histological analysis. This result suggested that the increased Cygb expression played a role in increasing the oxygen supply to support cell activity. High Cygb expression during the tissue regeneration process was necessary for meeting the oxygen demand of tissues [12]. According to Schmidt, the presence of Cygb protein in hypoxic conditions is needed to bind the oxygen from erythrocytes and carry it into the mitochondria [10].

The IHC analysis showed that Cygb proteins spread through various cells, such as the epithelial, erythrocyte, muscle, and neuronal cells. We therefore deduced that Cygb plays a role in supplying oxygen to the cells during the tissue regeneration of house gecko tails. In Schmidt’s study, Cygb protein appeared in fibrosis tissue in the hypoxia state [31]. Cygb protein has a strong oxygen-binding ability, like other globin proteins, such as the hemoglobin protein in blood cells, the neuroglobin protein in neuronal cells, and the myoglobin protein in muscles cells [10, 11]. Oxygen binding by Cygb continues with oxygen diffusion to the mitochondria. Cygb carries oxygen into the mitochondria to support the oxidative phosphorylation reaction, as well as the myoglobin functioning in muscle cells [12, 14]. The other function of Cygb is as an oxygen sensor, which regulates the activity of proteins in response to oxygen level changes in tissues [32]. Cygb is also involved in the synthesis of extracellular matrix proteins, such as fibroblasts, chondroblasts, and osteoblasts all synthesize collagen. Cygb contributes to lipid cell signaling, which increases antioxidants and protects against oxidative stress [14, 32, 33]. A study of Spalax rats found that Cygb spread in the neuronal cells and fibroblasts [8]. Singh’s study proved the role of Cygb in the regeneration of skeletal muscle, because it was found in the nucleus of basal lamina cells during the regeneration process of skeletal muscles. They found that the Cygb gene knockdown increased the number of dead cells in HeLa and COS cells. Cygb depletion also had an impact on the process of cell proliferation and differentiation that inhibited cell maturation in the myotubes of mice. In addition, Cygb deletion inhibited the repair and regeneration of muscle tissue in vivo [32]. The Halligen research results showed that reduced levels of Cygb in murine cell lines caused inhibition of cell proliferation by up to 70% and, when Cygb levels returned to normal, the inhibition decreased to 55% [31].

We suggest that, after Cygb distributes oxygen to the mitochondria, cells are activated to proliferate, migrate, and differentiate. The need for oxygen is high, but not accompanied by an increase in adequate oxygen supply to the tissues, placing the tissues in a state of relative hypoxia. According to Gauron, an imbalance in oxygen demand and oxygen supply causes tissues to become hypoxic [4]. Cygb can store oxygen and release it under hypoxic conditions, [9, 12] so we suggest that Cygb is involved in the adaptive response to injury.

High energy levels are needed to support cell activity in tissue regeneration [4, 7].

During tissue regeneration, the PGC-1α gene expression was lower than the Cygb gene expression; however, the PGC-1α expression increased and reached a peak earlier than Cygb. We suggest that the tissue tried to increase the number of mitochondria before Cygb supplied oxygen to them. High PGC-1α and Cygb expressions during tissue regeneration indicated the high energy demand. According to Jornayvaz, the PGC-1α protein plays a role in stimulating mitochondrial biogenesis, resulting in an increase of energy production [18]. Osama’s study showed that glycolysis increased during the regeneration of Planaria tissue [7].

The increased PGC-1α expression indicated the high activity level of mitochondrial biogenesis, and we suggest that this increase of PGC-1α expression related to cell activity. Wright et al. [19] showed the role of PGC-1α in cell proliferation and cell growth in cancer cells. Birkett et al. reported that overexpression of PGC-1α induced mitochondrial proliferation in rat liver and a cardiomyocyte cell culture [34]. Knockout of the PGC-1α and PGC-1β genes in the hearts of mice results in death in newborn mice. A study of human cardiac cells demonstrated the role of PGC-1α in heart cell proliferation [34]. Expression of the PGC-1α gene decreased in the second phase of regeneration of the house gecko tail tissue, but remained higher than that of the control group until the last day of observation.

The synergy of the Cygb and PGC-1α expressions showed an interesting pattern, with PGC-1α expression reaching its peak earlier than Cygb. The expression curves of these two genes indicated that the tissue was trying to meet the need for a certain number of mitochondria the cells before Cygb could plays its role in supporting oxygen diffusion to the mitochondria. The peak expressions of PGC-1α and Cygb occurred in the first phase (the wound healing phase), indicating that this phase required high levels of oxygen and energy. Cell migration, proliferation, and differentiation occurred to prepare the cells to enter the regeneration phase after the wound healing phase and stimulate the expression of Cygb and PGC-1α to meet the associated oxygen and energy requirements.

The dynamics of Cygb and PGC-1α gene expression in the regeneration of house gecko tail tissue showed that both of these genes play role in tissue regeneration. We suggest that the process of tissue regeneration depends on the energy produced in the mitochondria, supported by an adequate supply of oxygen. We hope that the results of this study can be applied to tissue regeneration therapy in humans. Further research on the role of the Cygb and PGC-1α knockout genes in the regeneration of house gecko tails will strengthen our hypothesis of the role of these two genes in the tissue regeneration process.

## Conclusion

The high expression of the Cygb gene during tissue regeneration indicated that the tissue regeneration process needed the oxygen bound by the Cygb. The high expression of PGC-1α at the beginning of tissue regeneration indicated that the mitochondrial biogenesis process occurred at this stage. We therefore assumed that the Cygb and PGC-1α genes played a role in the tissue regeneration process of house gecko tails.

## Methods

We used a healthy house geckos, as indicated by their normal animal activity, body weight, anatomy, and skin color. Healthy subjects were identified by their ability to successfully catch their prey. The physical characteristics of the animals were recommended by a herpetologist from the Zoology Laboratory at the Indonesian Institute of Sciences (LIPI): Dr. rer. Nat. Evy Ayu Arida.

The research method used descriptive-analytical experimental and cohort design to analyze the tissue regeneration of the house gecko tails. The source of the research animals was the Zoology Laboratory of the Indonesian Institute of Sciences (LIPI), Cibinong, Indonesia. The research procedures included animal adaptation, an autotomization procedure, and use of a DNA primer design, RNA isolation, qPCR, hematoxylin and eosin staining, western blot, and IHC for the data collection and statistical analyses of the data. Ethical permission for the research was obtained from the Faculty of Medicine of the University of Indonesia’s (FKUI’s) Research Ethics Committee, with no. 672/UN2.F1/ETIK/VII /2.

The research was performed in the Zoology Laboratory of the Indonesian Institute of Sciences (LIPI), Cibinong, Indonesia; the laboratory of the Histology Department, Faculty of Medicine, Universitas Indonesia; and the laboratory of the Center of Hypoxia and Oxidative Stress Studies in the Department of Biochemistry & Molecular Biology, Faculty of Medicine, Universitas Indonesia, from January 2015 to February 2018. All the animal research procedures were covered by Animal Scientific Procedures licenses.

### Materials and reagents

The materials and reagents used in our research included Cygb primary antibody obtained from Rabbit polyclonal anti-Cygb (Life-Span BioSciences LS-C312809) and anti-mouse IgG secondary antibody obtained from Trekkie Universal Link in Starr Trek Universal HR Detection System (BioCare Medical STUHRP700 H). The isolation of RNA was carried out using a MasterPure™ RNA Purification KIT) Epicenter Illumina Company, Water-Biotechnology). The gene expression was analyzed using a KAPA SYBR FAST one-step RT qPCR kit (Universal Kapa Biosystems, KK4650) and the machine for analyzing the qPCR was an Eco48 from Illumina.

### Animal model

The characteristics of the house gecko as the animal model included body weight (5 ± 0.5 g), body length (10–13 cm), and tail length (3.8–4.0 cm; i.e., a tail length less than half of the body length). The International Classification of *Hemidactylus platyurus* includes the Reptilia class, the Squamata order, the Gekkonidae family, and the Hemidactylus genus [22, 35]. Thirty three house geckos were kept in a glass cage with a size of 40 × 20 × 30 cm3 and adapted for 1 week at the Zoological Herpetology Laboratory, LIPI. The house gecko cage was exposed to sunlight in a room, with a variation of 12 h of light and 12 h of darkness. The geckos were fed with small live insects, such as mosquitoes, cockroaches, and grasshoppers, and were given water ad libitum in a small bowl placed in the middle of the cage.

The number of animal models, based on Federer’s formula, was thirty-three, divided into 10 experimental groups and 1 control groups. Each group consisted of three geckos.

Federer’s formula: (t - 1) (r – 1) ≥ 15.

*t* = experimental groups and control (11 groups).

*r* = the number of animals in each experimental group.

(11–1) (r – 1) ≥ 15, *r* = 3 animals/group.

### Autotomy procedure

All the house geckos were chosen randomly for autotomization, leaving non-autotomized house geckos as the control group. The house geckos release their tails naturally. The procedures for the autotomization accorded with the protocol of the Herpetology Laboratory, LIPI. The ten autotomized house gecko groups were returned to the cage and allowed to regenerate their tails until days 1, 3, 5, 8, 10, 13, 17, 21, 25, and 30, respectively.

### Tissue collection and analyses

The house geckos that had regenerated their tails, and the control group, were euthanized on days 1, 3, 5, 8, 10, 13, 17, 21, 25, 30, respectively, using an intravenous injection of ketamine (100 mg/mL) with a dosage of 10 mg/kg BW and xylazine (20 mg/mL) with dosage of 2 mg/kg BW. For house geckos weighing 5 g, a mixture of ketamine and xylazine was administered in 0,5 uL volume .

**Fig. 5:**
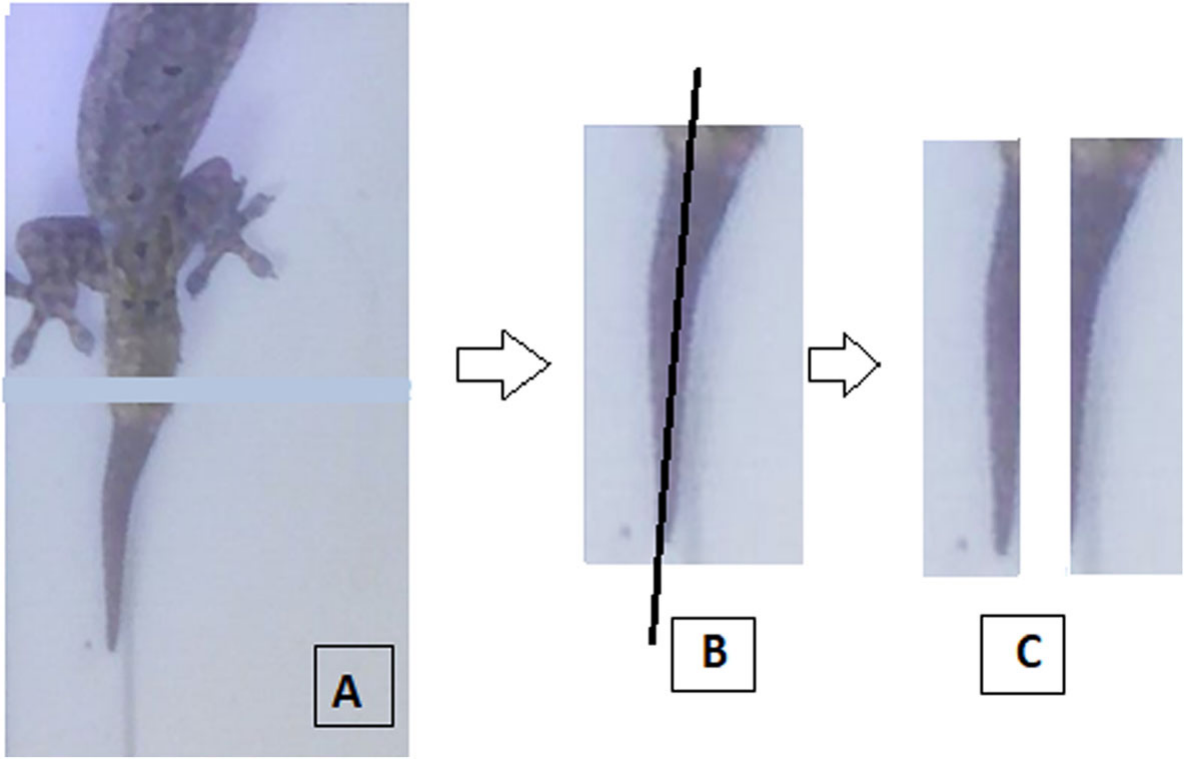
Sample preparation from house gecko (*Hemidactylus platyurus*) tails: **a** regenerated house gecko tail after autotomy, cut at the base of the regenerated tail in cross section for sample preparation; **b** longitudinal sectioning of regenerated tail for sample analysis; **c** the first part was used for histological analysis HE and IHC staining, and the other parts for the qPCR and western blot analyses for the same organism

The regenerated tail tissue was cut in cross section using a size 11 scalpel proximal to the injury site (Fig. 5a). The analysis for each time point used a different set of animals. Each sample of tail regenerated from each individual was used for the histological analysis, IHC analysis, qPCR analysis, and western blot analysis (Fig. 5b-c) The sample was cut into longitudinal sections; the first part was used for the histological analysis (HE and IHC staining) and stored in formalin 70%, while the other sections were used for the qPCR analysis and western blot analysis, stored in RNAse solution for analyzing the gene expression and at − 80 °C for analyzing the protein expression.

The gene expressions of Cygb and PGC-1α were analyzed using qPCR. The Cygb protein expression was analyzed using IHC and western blot, and the histological analysis used hematoxylin-eosin staining.

### Design of Primer DNA

Tracking of the Cygb, PGC-1α, and 18S ribosome (housekeeping) genes of the house gecko (*Hemidactylus platyurus*) began with phylogenetic study of the species with the closest taxonomic kinship to *Hemidactylus platyurus*. *Gesscko* is taxonomically the closest species to *H. platyurus*. The *Gecko* genome was described and deposited with the National Center for Biotechnology Information (NCBI) at https://www.ncbi.nlm.nih.gov/. These genes were analyzed with the BLAST method to find the sequences of the genes. A determination of DNA-conserved sequences was made using multiple alignments with Clustal X in the Mega7 software, and the primer DNA was designed using the Primer3 software.

### Isolation of total RNA

The frozen regenerated tail tissue was crushed using a micro-homogenizer, and the isolation of total RNA was carried out using the Illumina Company’s Epicentre MasterPure™ RNA Purification Kit (www.epibio.com/applications/nucleic-acid.kits/rna/masterpure-rna-purification-kit). The isolated RNA was pipetted into a 35 μL solution of TE buffer and stored at -80 °C.

### Quantification PCR (qPCR)

The RNA was converted to cDNA using the KAPA SYBR FAST RT-qPCR reverse transcription system for the cDNA that was used as a template for the qPCR reactions. The expression of Cygb and PGC-1α mRNA were determined using the Livak formula, with 18S RNA as a housekeeping gene.

### **Hematoxylin** a**nd eosin (HE) staining**

Regenerated tail tissues from days 1, 3, 5, 8, 10, 13, 17, 21, 25, 30 after autotomy, and tissues from the control group, were stored in formalin overnight. The tissues were dehydrated with alcohol for 24 h and subsequently purified with xylol for 24 h. The tail tissues were then embedded in liquid paraffin and left to solidify into block paraffin to be cut by machine with a thickness of 4–5 μm. The sample slices were mounted on glass objects and incubated for 24 h. The slices were then ready to be stained with hematoxylin-eosin.

### Immunohistochemistry

IHC analysis was performed on the paraformaldehyde-fixed and paraffin-embedded samples. We used rabbit antibody anti-Cygb (MyBioSource and LifeSpan BioSciences) 1:1000 as the primary antibody and Trekkie Universal Link (BioCare Medical) as the secondary antibody. HRP streptavidin was used as a probe that bound to the secondary antibody. HRP was detected by DAB-Chromogen dye and visualized by ImageQuant™.

### Quantitative histological analysis by ImageJ I-46 program

**Fig. 6:**
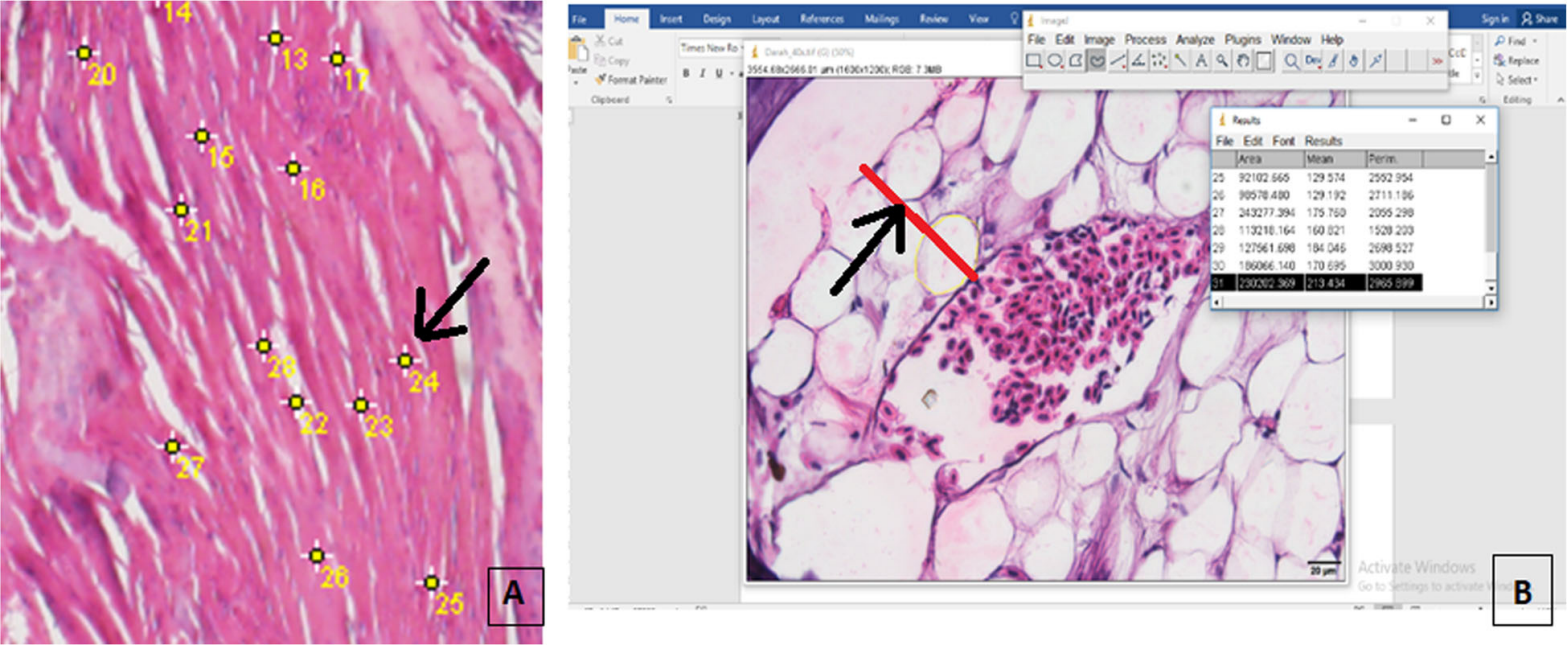
The Image J I-46 program for the quantification of the histological analysis: **a** the cell numbering, with the cell marked by the number (black arrow); **b** the red line pointed to by the black arrow showing the width of the tissue, at a magnification of 40 × 10

The Image J I-46 program was used to calculate the number of cells and measure the length or width of the tissue samples. (Fig. 6a). For the length or the width of the tissue area, we set the scale for the image using the function in the Image J I-46 program, and the results of the measurements appeared as lines, automatically, in the feature (Fig. 6b).

### Data collection

The data for the Cygb and PGC-1α (mRNA) gene expressions was analyzed. The data for the Cygb protein expression, the tail growth length, and the histology analyses was all collected from the same individual and subjected to statistical analysis.

### Statistical analyses

The data distribution was analyzed with a Kolmogorov-Smirnov test. If the data distribution was normal, a comparative analysis was carried out for every growth day group using the one-way ANOVA; if the data distribution was not normal, the comparative analysis for every group was carried out using the Kruskal-Wallis test. The differences for every group were considered to be significant at the *p* value < 0.05.

## Supplementary information

**Additional file 1: Table S1.** Post hoc test of varied data of Cygb mRNA between each growth-day group (ANOVA, *p* < 0.05). **Table S2.** Man-Whitney test (*p* value) for each group between groups for PGC-1α. **Table S3.** The results for primer DNA of Cygb, PGC-1α, and 18S genes designed by using multiple alignment

## Data Availability

The data sets used analysed during this current study available from the corresponding author on reasonable request.

## Abbreviations

CYGB: Cytoglobin
PGC-1α: Peroxisome proliferator-activated receptor gamma coactivator-1α
qPCR: Quantitative polymerase chain reaction
Nrf-1: Nuclear respiratory factor-1
mtTFA: Mitochondrial transcription factor-A
HE: Haematoxylin-Eosin
HRP: Horse Radish Peroxidase
