# Supplementary material for: High expressions of the cytoglobin and PGC-1α genes during the tissue regeneration of house gecko (Hemidactylus platyurus) tails
Source: BMC Dev Biol. 2020 May 11;20:11. doi: 10.1186/s12861-020-00214-4 (PMC7216616; doi:10.1186/s12861-020-00214-4)
Supplement: Supplementary file 1 — Additional file 1: Table S1. Post hoc test of varied data of Cygb mRNA between each growth-day group (ANOVA, p < 0.05). Table S2. Man-Whitney test (p value) for each group between groups for PGC-1α. Table S3. The results for primer DNA of Cygb, PGC-1α, and 18S genes designed by using multiple alignment [file 12861_2020_214_MOESM1_ESM.docx]

Table 1. The results for primer DNA of Cygb, PGC-1α, and 18S genes designed by using multiple alignment

| Gene | Forward | Reverse | End product |
| --- | --- | --- | --- |
| Cygb | 5'-CTC CTC TGT ACT GGC CTT GG-3' | 5'-CTC CTC TGT ACT GGC CTT GG-3' | 198 bp |
| PGC-1α | 5'-TCT CGA TCG GGA ATATGG AG -3' | 5'- GAT CTG TCG CCT TCT TGC TC -3' | 159 bp |
| 18S ribosome | 5'-ACA CGC TCC ACC TCA TCT TC-3' | 5'-ATC CCA GAG AAG TTC CAG CA-3' | 188 bp |

Table 2. ANOVA Post hoc test of varied data of Cygb mRNA between each growth-day group (p < 0.05)

|  | Day-1 | Day-3 | Day-5 | Day-8 | Day-10 | Day-13 | Day-17 | Day-21 | Day 25 | Day 30 |
| --- | --- | --- | --- | --- | --- | --- | --- | --- | --- | --- |
| Day-1 |  | .216 | .112 | .012 | .011 | .036 | .387 | .729 | .844 | .436 |
| Day-3 |  |  | .072 | .151 | .145 | .341 | .702 | .368 | .157 | .052 |
| Day-5 |  |  |  | .282 | .272 | .564 | .447 | .206 | .078 | .023 |
| Day-8 |  |  |  |  | .982 | .609 | .074 | .025 | .008 | .002 |
| Day-10 |  |  |  |  |  | .594 | .071 | .024 | .007 | .002 |
| Day-13 |  |  |  |  |  |  | .188 | .073 | .024 | .006 |
| Day-17 |  |  |  |  |  |  |  | .600 | .291 | .108 |
| Day-21 |  |  |  |  |  |  |  |  | .588 | .265 |
| Day-25 |  |  |  |  |  |  |  |  |  | .558 |

Table 3. Man-Whitney test post hoc (p value) for each group between groups for of

varied data of PGC-1α mRNA

|  | Day-1 | Day-3 | Day-5 | Day-8 | Day-10 | Day-13 | Day-17 | Day-21 | Day-25 | Day-30 |
| --- | --- | --- | --- | --- | --- | --- | --- | --- | --- | --- |
| Day-1 |  | 0.05 | 0.05 | 0.05 | 0.05 | 0.05 | 0.127 | 0.827 | 0.513 | 0.513 |
| Day-3 |  |  | 0.275 | 0.513 | 0.275 | 0.05 | 0.05 | 0.05 | 0.05 | 0.05 |
| Day-5 |  |  |  | 0.513 | 0.127 | 0.05 | 0.05 | 0.05 | 0.05 | 0.05 |
| Day-8 |  |  |  |  | 0.05 | 0.05 | 0.05 | 0.05 | 0.05 | 0.05 |
| Day-10 |  |  |  |  |  | 0.127 | 0.127 | 0.05 | 0.05 | 0.127 |
| Day-13 |  |  |  |  |  |  | 0.827 | 0.275 | 0.05 | 0.05 |
| Day-17 |  |  |  |  |  |  |  | 0.275 | 0.127 | 0.275 |
| Day-21 |  |  |  |  |  |  |  |  | 0.827 | 0.513 |
| Day-25 |  |  |  |  |  |  |  |  |  | 0.513 |
